# Supplementary material for: 3-Hydroxy-3-Methylglutaric Acid Disrupts Brain Bioenergetics, Redox Homeostasis, and Mitochondrial Dynamics and Affects Neurodevelopment in Neonatal Wistar Rats
Source: Biomedicines. 2024 Jul 15;12(7):1563. doi: 10.3390/biomedicines12071563 (PMC11274636; doi:10.3390/biomedicines12071563)
Supplement: Supplementary file 1 [file biomedicines-12-01563-s001.zip › biomedicines-3038559-supplementary.pdf]

3-Hydroxy-3-methylglutaric acid disrupts brain bioenergetics, redox homeostasis, and mitochondrial dynamics and affects neurodevelopment in neonatal Wistar rats

Josyane de Andrade Silveira[1], Manuela Bianchin Marcuzzo[1], Jaqueline Santana da Rosa[1], Nathalia Simon Kist[1], Chrístofer Ian Hernandez Hoffmann[1], Andrey Soares Carvalho[1], Rafael Teixeira Ribeiro[1], André Quincozes-Santos[1,2,3], Carlos Alexandre Netto[1-4], Moacir Wajner[1,2,5], Guilhian Leipnitz[1-4*]

[1] Programa de Pós-Graduação em Ciências Biológicas: Bioquímica, Universidade Federal do Rio Grande do Sul, Rua Ramiro Barcelos, 2600-Anexo, Porto Alegre, RS 90035-003, Brazil; josysilveira94@gmail.com, manuelamarcuzzo9@gmail.com, jaquelinesantanarosa@gmail.com, nathaliakist@gmail.com, christoferianhh@gmail.com, andreyscarvalho@outlook.com, rafaelrated@hotmail.com, andrequincozes@ufrgs.br, netto@gabinete.ufrgs.br, mwajner@ufrgs.br, guilhian@ufrgs.br

[2] Departamento de Bioquímica, Instituto de Ciências Básicas da Saúde, Universidade Federal do Rio Grande do Sul, Rua Ramiro Barcelos, 2600-Anexo, Porto Alegre, RS 90035-003, Brazi; andrequincozes@ufrgs.br, netto@gabinete.ufrgs.br, mwajner@ufrgs.br, guilhian@ufrgs.br

[3] Programa de Pós-Graduação em Neurociências, Universidade Federal do Rio Grande do Sul, Rua Ramiro Barcelos, 2600-Anexo, Porto Alegre, RS 90035-003, Brazil; andrequincozes@ufrgs.br, netto@gabinete.ufrgs.br, guilhian@ufrgs.br

[4] Programa de Pós-Graduação em Ciências Biológicas: Fisiologia, Universidade Federal do Rio Grande do Sul, Rua Ramiro Barcelos, 2600-Anexo, Porto Alegre, RS 90035-003, Brazil; netto@gabinete.ufrgs.br, guilhian@ufrgs.br

[5] Serviço de Genética Médica, Hospital de Clínicas de Porto Alegre, Rua Ramiro Barcelos, 2350, Porto Alegre, RS 90035-903, Brazil; mwajner@ufrgs.br

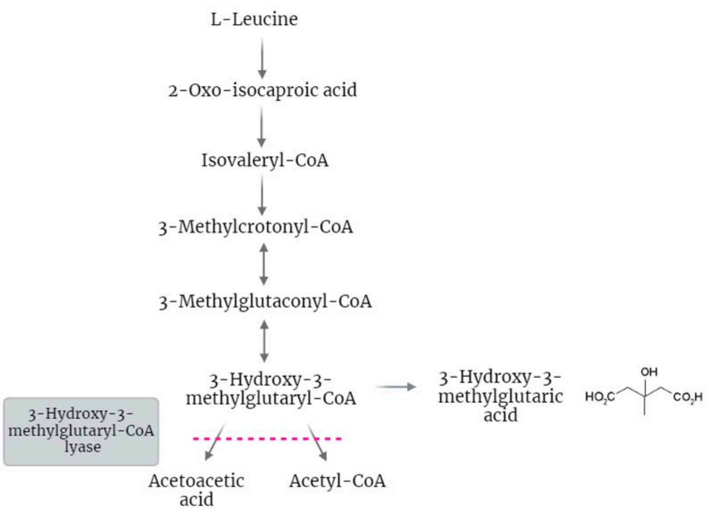

Supplementary Figure S1. Metabolic route of leucine catabolism and ketogenesis showing 3-hydroxy-3-methylglutaryl-CoA lyase deficiency

Supplementary Table S1. Number of animals used in each experimental group for evaluating the biochemical parameters and neurodevelopment

Parameters	Number of rats in Control group	Number of rats in test group
Antioxidant enzyme activities and reduced glutathione levels	5	5
Citric acid cycle enzyme and respiratory chain complex activities	5	5
Western blotting	6	6
Neurodevelopment markers	6	6
